# Non-invasive motor unit analysis reveals specific responses during maximal muscle contraction under normobaric hypoxia

**DOI:** 10.1007/s00424-025-03119-y

**Published:** 2025-09-10

**Authors:** Danilo Bondi, Giacomo Valli, Carmen Santangelo, Salvatore Annarumma, Tiziana Pietrangelo, Stefania Fulle, Vittore Verratti

**Affiliations:** 1 Department of Neuroscience, Imaging and Clinical Sciences, University “G. d’Annunzio” Chieti-Pescara, Via Dei Vestini, 31, 66100 Chieti, Italy; 2 Department of Clinical and Experimental Sciences, University of Brescia, Brescia, Italy; 3 Department of Biomolecular Sciences, University of Urbino, Urbino, Italy; 4 Department of Science, University “G. d’Annunzio” Chieti-Pescara, Chieti, Italy

**Keywords:** Simulated altitude, Conduction velocity, Discharge rate, High-density surface electromyography, Maximal voluntary contraction

## Abstract

Hypoxia has been extensively studied as a stressor which pushes human bodily systems to responses and adaptations. Nevertheless, a few evidence exist onto constituent trains of motor unit action potential, despite recent advancements which allow to decompose surface electromyographic signals. This study aimed to investigate motor unit properties from noninvasive approaches during maximal isometric exercise in normobaric hypoxia. Applying a cross-over design, 18 participants (gender-matched, on average age 22.6 y, BMI 23.6 kg/m^2^, and bioimpedance phase angle 6.4) were exposed twice to hypoxia (FiO_2_ ≊ 15.0% and FiO_2_ ≊ 13.4%, separately, by using a tent connected with a hypoxic generator) and once to normobaric normoxia. After ≊ 30 min inside the tent, participants performed a series of 9 unilateral isometric contractions of the right knee extensors at maximum intensity for 5 s, interspersed with 15 s of passive recovery, while acquiring high-density surface EMG signals through a 64-electrodes grid and cardiorespiratory variables, and registering symptoms; then, a post-processing motor unit decomposition technique was applied. We found an increase in MU discharge rate as a response to acute normobaric hypoxia, although to a little extent and differently across sexes. Moreover, males experienced a more prominent increase of MU conduction velocity due to hypoxia. MUs responses to normobaric hypoxia were only slightly and non-homogeneously associated with hypoxic cardiorespiratory responses. Normobaric hypoxia affects the neuromuscular system with a relatively greater effect on peripheral rather than central features.

**Supplementary Information:**

The online version contains supplementary material available at 10.1007/s00424-025-03119-y.

## Highlights

- Peripheral components, as expressed by conduction velocity, demonstrate greater sensitivity compared to central components, as expressed by motor unit firing rate, to maximal isometric exercise in normobaric hypoxia
- Neuromuscular and cardiorespiratory systems do not necessarily respond in a joint manner when triggered by hypoxic exercise
- Females exhibit reduced responsiveness to hypoxic stress in motor unit properties compared to males

**Supplementary Information:**

The online version contains supplementary material available at 10.1007/s00424-025-03119-y.

## Introduction

Hypoxia is a well-known stressor that pushes human bodily systems to respond and eventually adapt. Beyond clinics, environmental physiology has been focusing on normobaric and hypobaric hypoxia, and exercise physiology as well, since a fine tuning of hypoxic dose can burst benefit for further performance, e.g. through the stimulation of erythropoiesis and angiogenesis [1, 2]. Within this topic, a little subset of study has focused on neuromuscular function. Herein, a distinction is drawn between the central and peripheral properties of motor units. The discharge behaviour of the innervating motoneurons determines the central properties, which are expressed by metrics as recruitment and derecruitment threshold, and discharge rate; the morphology and biology of the innervated muscle fibres determine the peripheral properties, which are expressed by metrics as condition velocity and the amplitude of the action potentials [3].

The effects of high altitude-hypoxia on electrical and mechanical properties of muscle during isometric exercise at 20–100% MVC range did not influence electromyographic properties after 43 days, nor force output, nor motor unit sub-types ratio and activation pattern were affected during 40 days of high-altitude staying [4, 5]. For what concern the minimal altitude to see possible effects of hypoxia, alterations in muscle contractile properties can emerge already at 2,320 m of altitude [6]. For what concern the electromyography (EMG)-derived variables putatively responsive, by using surface electromyography (sEMG) in a normobaric hypoxia setting, (with fraction of inspired oxygen, i.e. FiO_2_, of 15% and 13%), no effect on root mean square (RMS) and mean frequency were found on *biceps brachii* [7]. Instead, setting FiO_2_ of 12.9% during leg extension at 70% MVC, EMG frequency was higher while amplitude was unaffected compared to normoxia [8]. With a more severe normobaric hypoxia (FiO_2_ = 10.8%), muscle activation of quadriceps at a given intensity was greater [9]. During maximal isometric bench press trials, normobaric hypoxia with FiO_2_ of 14.5% did not alter EMG amplitude with respect to normoxia [10]. Contrasting results exist on the role of hypoxia as a possible affecting-factor for muscle fiber conduction velocity [11–13]. Although fiber conduction velocity can remain unaltered, other differences such as higher excitability of α-motoneurons can emerge [14]. Normobaric hypoxia with FiO_2_ of 12% can reduce the resting motor threshold and increase cortical excitability [15]. However, another study reported an increase in resting motor threshold at high altitude [16]. Therefore, although it can be postulated that hypoxia increase fatigue during aerobic exercise, the influence of both acute and chronic hypoxia on peripheral and central fatigue during resistance exercise remains not homogeneously reported.

Maximal voluntary contraction and evoked compound muscle action potential are preserved in healthy humans exposed to both acute and chronic hypoxia [17], despite the greater central fatigue [18]. Impairment of muscle function preferentially emerge during sub-maximal intermittent contractions [17]. Ruggiero and colleagues investigated the effect of acclimatization to hypoxia, thereby reporting that acute hypoxia has detrimental affect on muscular function and that chronic hypoxia increase excitability of the motoneurons; they conclude the greater motoneuron responsiveness during fatiguing exercise may be an adaptive mechanism to restore performance [19]. Goodall and colleagues pointed out another potential adaptive mechanism: the greater corticospinal excitability in chronic hypoxia may reduce the development of exercise-induced supraspinal fatigue [20]. Comprehensively, acute hypoxia increase neuromuscular fatigability, whose supraspinal component is restored with acclimatization, as well as peripheral component but only if limited muscle groups are involved [21]. Herein we intended fatigue as the exercise-induced decline in neuromuscular performances, whose objective measurement is usually termed as fatigability. Neuromuscular fatigability analyzed through the lens of advanced EMG techniques and strength measurement allows to distinguish between peripheral, spinal and supraspinal components. Under these principles, it emerges the possibility that motor unit (MU) firing rate may be affected by the combined stressors of exercise and hypoxia. Indeed, acute hypoxia affects MU firing rate during isometric contractions, but the direction of changes, either increase or decrease, may be an individual-specific response; however, in the same work authors reported that those who experienced a faster reduction in SpO_2_ manifested the reduced firing rate [22]. Therefore, some missing links may exist in concomitant changes in neuromuscular and oxygen delivery system due to hypoxic exercise.

Little is known about the MU behavior as an acute response to hypoxia. We previously investigated sEMG data during an isometric squat at high vs low altitude, reporting that myoelectric features such as frequency and fiber conduction velocity were not affected; the unexpected slight decrease in root mean square we found was putatively attributed to preventive mechanism compensating for a relatively greater effort during a fatiguing strength test involving large muscle masses, or by a cumulative exertional stress on muscles after the high-altitude trek [23]. The effects of hypoxia on muscle fiber conduction velocity has not been fully clarified, and the use of MUs estimates from high-density sEMG setting would help to deep into this argument. Although questioned [24], we speculated that hypoxia per se may have minimal, if any, effects on the myoelectric activity of large muscle masses but, due to the field condition coupled with the type of task, the signals we obtained did not allow adequate estimation of single MU contributions [25]. During field studies, the occurrence of peripheral and central fatigability may alter myoelectrical response to hypoxia.

Mild hypoxia may not result in EMG alterations; however, most results come from upper arm muscles and non-maximal exercise. Moreover, high-density set-up that allow decomposition of single MU contributions are at their dawn in environmental physiology, in spite of technological and analytical advances that currently allow a larger use of High-Density surface EMG (HDsEMG) to depict better the neuromuscular response adaptations to environmental stressors. We hypothesized that maximal isometric exertion involving isolated muscle mass could stimulate the occurrence of central and peripheral effects in healthy young adults as hypoxia intensifies, as detectable by HDsEMG procedures.

### Aims

This study used a normobaric hypoxia experimental set-up to disentangle central and peripheral features of neuromotor response during maximal muscle contractions by taking advantage of non-invasive methods, while avoiding the contextual factors as cold and physical activity usually present in altitude hypoxia studies. With this study, we aimed to investigate non-invasively how MU properties change while performing isometric exercise at different degrees of normobaric hypoxia. In addition, we aimed to verify whether cardiorespiratory responses influence hypoxic-induced MUs changes.

## Methods

### Study design

This study came with a single-blind crossover design, which consisted of three conditions with different FiO_2_, spaced ≊ 7 days apart from one session to another. One condition served as control, thus applying normoxia by maintaining FiO_2_ ≊ 20.9%. Participants were randomly assigned an administration order and remained unaware of such order until the completion of all sessions. Two conditions served as normobaric hypoxia, the one achieved with FiO_2_ ≊ 15.1% (which corresponds to hypoxia at an altitude ≊ 2,500 m a.s.l.) and the other achieved with FiO_2_ ≤ 13.6% (which corresponds to hypoxia at an altitude ≥ 3,500 m a.s.l.). The project involved other two study branches, focused on associative memory [26] and network physiology (Bondi, Morandotti et al*.*, article under review).

### Participants

Participants with the following medical conditions were excluded from the study: current diagnosis of ischemic heart disease, including coronary artery disease and angina pectoris; past acute myocardial infarction; chronic obstructive pulmonary disease; psychiatric disorders such as psychosis, neurosis, schizophrenia, depression, alcoholism, substance abuse, and neurodegenerative diseases; neurological disorders; respiratory failure diagnosis; uncontrolled hypertension (diastolic > 95 mmHg and/or systolic > 180 mmHg); individuals undergoing anticoagulant therapy.

For obtaining a large effect size (η^2^_p_ = 0.14), with statistical significance (α error probability) of 0.05 and statistical power (1–β) of 0.9, i.e. 90%, estimating correlation among repeated measures in a within factors design with 3 repeated measures of 0.5, a minimum sample size of 15 participants was calculated (calculations carried out using software G*Power v. 3.1.9.3). Accounting for a possible drop-out of 10% and a gender-balanced recruitment, a total of 18 healthy students, of which nine females and nine males, aged from 20 to 29 years (mean of 22.6 y, SD of 2.74 y) voluntarily took part in the study, during the Physiology course in the *Medical Degree Program* at the University "G. d'Annunzio" Chieti-Pescara, Italy.

All participants provided their written informed consent after being informed about risks of the study, which was approved by the local ethical committee (Comitato Etico delle Province di Chieti e Pescara, document n. 18, 29/07/2021) and whose entire procedure was carried out in accordance with the ethical standards of the Declaration of Helsinki. Physical activity levels ranged from inactivity (7 participants) to sports participation (1 female non-professional volleyball player) with the other participants practicing fitness 2–4 times per week.

### Bioimpedance analysis (BIA)

The whole methodological section is detailed in the Supplementary Materials (S1). Briefly, body composition measurements were carried out by a specialist using the HUMAN IM TOUCH multi-frequency analyzer (DS MEDICA, Milan, Italy). By referring to the rationale of BIA [27, 28], to reference values [29–31] and to recommendations [32], we carried out BIA analysis as a surrogate of direct measures of skeletal muscle status of participants, thereby allowing to obtain information both on regional muscle features and on whole body metrics, which have been associated with strength [33, 34]. BIA analysis was reported to share the characteristics of the group under study, with a view to reproducibility and further comparison; no a priori exclusion criteria were applied based on the results to ensure adherence to the anthropometric status representative of the young population. Data obtained from BIA are shown in Table 1. One male participant showed particularly high BMI (35.1 kg/m^2^), usually class II obesity, but attributed to high muscularity due to his advanced level of fitness. Phase angle was found in line with the reference cut-off for both sex.

**Table 1:**
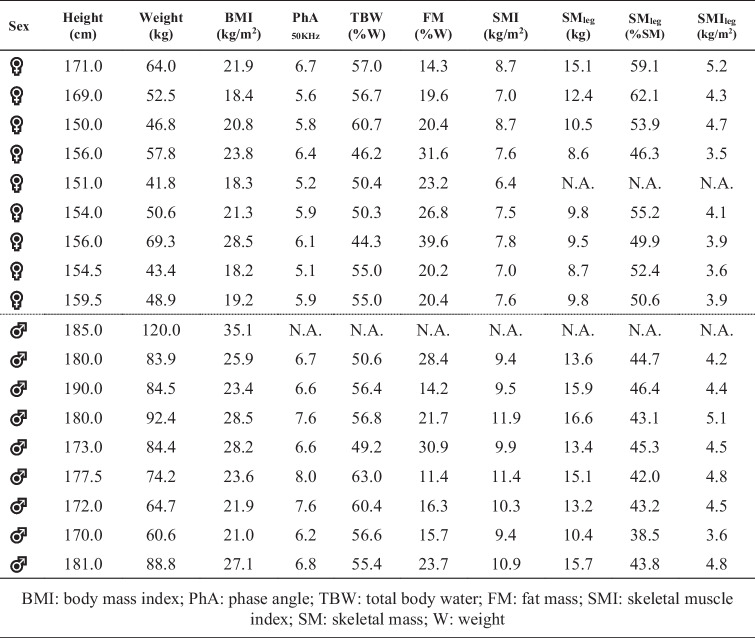
Report of bioimpedance analyses

*BMI* Body mass index; *PhA* Phase angle; *TBW* Total body water; *FM* Fat mass; *SMI* Skeletal muscle index; *SM* Skeletal mass; *W* Weight

### Procedures

Participants were individually tested in a tent coupled with an altitude generator (*Everest Summit II, Hypoxico, USA*), that allowed to reduce the oxygen content in the environment while maintaining constant atmospheric pressure (i.e. normobaric hypoxia). During tests, FiO_2_ was frequently checked by using the integrated oxymeter (*Handi oxygen monitor, Hypoxico, USA*) and adjusted. Participants remained in the tent for approximately 30 min. The duration was set in order to allow an exposure time ≥ of the 20-min hypoxic challenge test (HCT), which is commonly used to assess hypoxemia during approximately comparable inspired oxygen tension to breathing air at 2,438 m [35]. The experimental procedure included a rest phase for participant acclimatization (10 min), an associative memory task (15–20 min), and the physical task (3 min) consisting of a series of 9 unilateral isometric contractions of the right knee extensors at maximum intensity for 5 s, interspersed with 15 s of passive recovery. The number and duration of isometric contractions and exercise phases were heuristically set after preliminary trials, starting with lower numbers and different times, and consecutive EMG analysis. The absence of a linear up phase and a controlled steady state phase, along with relatively complex architecture of *vastus lateralis* and the presence of subcutaneous fat limit MUs decomposition performance. Despite the above-mentioned limitations, we applied preliminary quality control and gold-standard analytical procedures in order to estimate MUs behaviour during maximal contractions, which are more representative of real-life contraction. After each session, participants reported subjective symptoms, and they were asked to note any delayed symptoms in the hours and days following.

### Cardiorespiratory variables

The whole methodological section related to metabolic cart is reported elsewhere (Bondi, Morandotti et al*.*, article under review). Briefly, the K5 Wearable Metabolic System (COSMED srl, Roma, Italy) was used in breath-by-breath (B × B) mode to register heart rate (HR), ventilation (V̇e), and oxygen consumption (V̇O_2_), whose individual series were filtered by removing outliers, fitting and interpolating. Peripheral saturation (SpO_2_) was registered with a finger pulse oxymeter (Beurer PO80, Beurer GmbH, Germany) at the beginning (outside the tent) and at the end (inside the tent) of each condition. Before each session, all calibration routines were performed according to the manufacturer’s instructions.

### High-density surface electromyography (HDsEMG)

All the procedures were carried out following current recommendations for signal acquisition, positioning of grids, and settings for the analyses of both central and peripheral MU properties from decomposed signals [3, 36, 37].

Due to the inability of fitting a dynamometer chair in the hypoxic tent to control a voluntary contraction close to MVC in a steady state phase, EMG signals were recorded during maximal contractions. To perform the maximal contractions, participants were made to sit on a sturdy couch with their hip and knee at 90° and their hip and ankle secured to the chair with well-anchored straps. The participants were requested to reach their maximum knee extension force within 1 s and to maintain the contraction for a total of 5 s. During the maximal isometric tasks, HDsEMG was acquired from the vastus lateralis muscle of the dominant (right) limb, with one semi-disposal adhesive grid of 64 (over 13 rows × 5 columns) gold-coated electrodes of diameter 1 mm with inter-electrode distance of 8 mm (GR08MM1305, OT Bioelettronica, Torino, Italy). The skin was prepared by abrasion and cleaning. The grid was fixed to the skin by using bi-adhesive perforated foam layers (SpesMedica, Battipaglia, Italy), whose cavities were filled with a conductive paste (SpesMedica) and by medical tape to minimize movement artifacts.

Positioning of the grid was standardized by making note of the distal innervation zone in the vastus lateralis for each participant [38]. This identification was carried out outside the tent, while the participant was sitting before the first trial started. In particular, the following procedure was applied: 1) identifying the muscle by observation and palpation, 2) hypothesizing the location of distal motor points, 3) using a pen-electrode and a reference electrode of a neuromuscular electrical stimulation (NMES) device (Genesy 1200 Pro; Globus Srl, Italy) to find the point of greatest mechanical response per current dose (i.e., motor point, MP) by moving the pen-electrode to adjacent locations. In particular, MP identification relies on the fact that muscle displacements induced by the contractile response is clearly observed even at very low stimulation intensities when the pen is facing the MP area. The placement of the grid was directed over the contracting muscle belly to ensure complete adherence of the grid to the muscle belly, even among participants with less developed muscles. Strap electrodes dampened with water were placed around the ipsilateral (reference electrode) and contralateral (ground electrode) ankle. An expert operator conducted all the acquisitions. The centre of the grid and the orientation were acquired for each participant by using a tape measure and a goniometer, and registered; this was done in order to maintain the same positioning across conditions while avoiding the use of permanent markers.

The quality of EMG signals was preventively checked by quantifying the noise level through estimation of resting RMS (to be lower than 20 μV). Raw EMGs were registered in monopolar configuration with a sampling frequency of 2048 Hz, filtered with bandpass 10–500 Hz, and converted to digital data using a multichannel amplifier (EMG-Quattrocento; OT Bioelettronica). Signals were recorded and further visualized with the software OT Biolab + v1.5.6 (OT Bioelettronica) and exported for MU decomposition.

### Motor unit analysis

For the scope of this study, only the first 5 maximal contractions were considered to avoid muscle fatigue. Of these, the 2 contractions with the greatest EMG signal amplitude (root mean square) have been selected for signal decomposition and MU detection.

MU decomposition has been performed with a validated multichannel blind source separation technique [39] on the 2 selected maximal contractions after band-pass filtering the EMG signal between 20 and 500 Hz (2nd order, Butterworth). This decomposition technique has been successfully adopted to decompose short-duration contractions in previous studies [40]. This pragmatic approach in our experimental setting was realized by adopting several inspections techniques as to obtain valid MUs estimation.

**Fig. 1:**
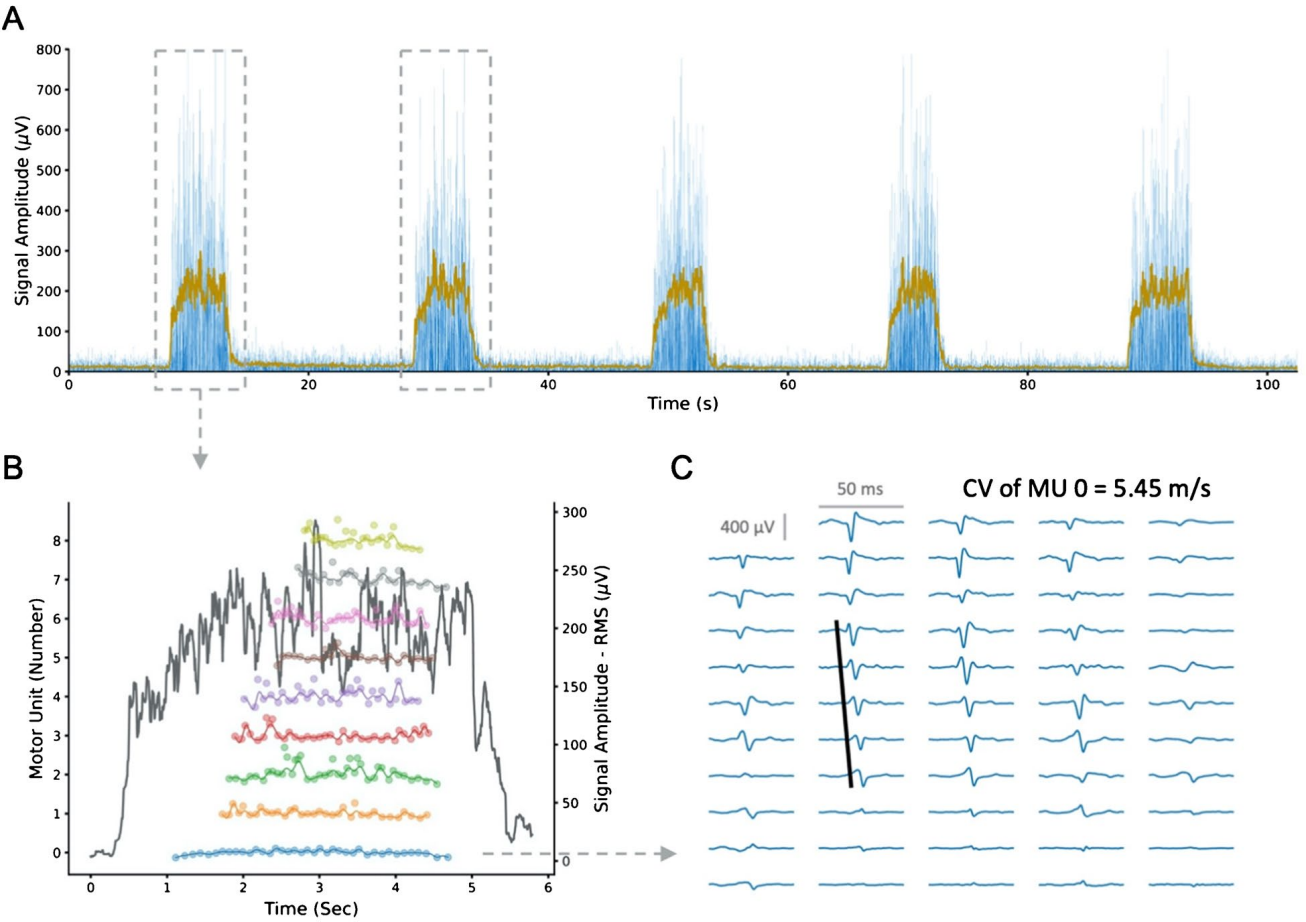
Schematic representation of High-Density surface Electromyography (HDsEMG) signal processing and analysis. **A** Among five consecutive maximal contractions, the two with the highest signal amplitude, determined by the average root mean square (RMS) across channels, were selected for signal decomposition and motor unit (MU) detection. **B** Instantaneous discharge rates were computed from the decomposed MU discharge patterns, and the mean smoothed MU discharge rate was calculated. **C** Conduction velocity (CV) was estimated from the best propagating MU action potentials

The detected MUs have been visually inspected and manually edited by an experienced operator. Only those MUs with a pulse to noise ratio above 28 have been considered for further analyses [41]. Given the absence of a reference signal, the manual editing phase was supported by the observation of the mean EMG root mean square as an indicator of the muscle activation level (Fig. 1).

From the detected discharge times, the instantaneous discharge rate (IDR) has been calculated [3]. A smooth estimate of MU’s IDR has been obtained by convolution of the IDR over a 4 discharges hanning window (method adapted from [42]). From the smoothed DR profile, the mean smoothed DR has been obtained. For the scope of this study, we preferred to maintain a cautious approach and to use a minimally smoothed IDR profile for the calculation of mean DR instead of the raw IDR, given that the complexity in the manual editing of maximal contractions may introduce non-physiological IDR values that are difficult to adjust.

MU action potentials (MUAP) have been obtained via spike-triggered averaging of all the discharge times in a 50 ms time-window on the double-differential derivation of the EMG signal computed across the grid’s columns [3]. MUs presenting clearly propagating MUAPs have been selected to estimate MU’s conduction velocity (CV). The largest number of channels suitable for MU’s CV estimation were manually selected by an experienced operator based on the clear propagation of the action potentials, a cross-correlation coefficient between adjacent channels above 0.8 and no innervation zone or end of fiber effect [43]. If multiple columns presented suitable MUAPs, the selection prioritized the column with the highest average cross-correlation coefficient [3]. On the selected channels, MU’s CV was calculated using a maximum likelihood estimation of delay [44]. MU decomposition and editing was done in MATLAB (R2023a; The Mathworks Inc., Natick, MA) with the DEMUSE software while all the analyses were performed in Python (V3.11.6, Python Software Foundation, USA) with the *openhdemg* V0.1.0 library [3].

### Data analysis

Hypoxic ventilatory response (HVR) and hypoxic cardiac response (HCR) were computed as follows, by adapting the equations reported by Richalet and colleagues [45]:

1 $$HVR=\frac{\left({\dot{V}e}_{(H)}-{\dot{V}e}_{(N)}\right)}{\Delta {SpO}_{2}}$$

2 $$HCR=\frac{\left({HR}_{(H)}-{HR}_{(N)}\right)}{\Delta {SpO}_{2}}$$

were V̇e is ventilation, ΔSpO_2_ is the difference in peripheral saturation (usually computed as SpO_2_ in normoxia – SpO_2_ in hypoxia), HR is heart rate, H refers to any hypoxic condition, and N refers to normoxia. HVR and HCR were computed both for identifying the response from normoxia to Hypoxia1, and from normoxia to Hypoxia2. Greater response refers to higher increase in V̇e or HR during hypoxia in spite of lower desaturation. Since in hypoxia desaturation is likely to occur in any individual, if increases in in V̇e or HR occur, results of HVR and HCR are numerically positive. The median values of V̇e and HR registered through the COSMED K5 during the exercise phase was used for computing HVR and HCR. We then computed the response of MUs variables and VO_2_ as a simple difference, non-normalized to desaturation, as follows:

3 $$\Delta CV={CV}_{(H)}-{CV}_{(N)}$$

4 $$\Delta IDR={CV}_{(H)}-{CV}_{(N)}$$

5 $${\Delta VO}_{2}={{VO}_{2}}_{(H)}-{{VO}_{2}}_{(N)}$$

To obtain summary values for these computation, the median values of CV and IDR across the different MUs identified for each participant were used. Median was used in spite of mean to deal with possible outliers.

### Statistics

To analyze the results of MUs properties (CV and IDR, separately), firstly normality of residuals was checked with Kolomogorov-Smirnov test and Q-Q plots observation. IDR data were filtered by removing outliers for *z*-scores beyond the threshold of ± 2.5 and by applying a logarithmic transformation. Then, a series of linear mixed model analysis fit by restricted maximum likelihood (REML) was carried out, setting hypoxia and sex as the fixed factors and individuals as the random factor. The level of significance was set for p < 0.05 and both the marginal and conditional R^2^ were reported. Satterthwaite method for degrees of freedom was used to compute effect size (through online tools available at https://effect-size-calculator.herokuapp.com); both models' features and different effect size were reported as to account for further comparisons. These analyses were carried out by using the GAMLj module (Gallucci, M. (2019). *GAMLj: General analyses for linear models*. [jamovi module]. Retrieved from https://gamlj.github.io/.) on software Jamovi (version 2.3.21.0). Post-hoc comparisons were carried out by accounting for multiple comparison correction with Holm method. Graphs of MUs properties show median and IQR, despite the use of parametric analysis for statistical comparisons, for facilitating the visualization of our results.

After checking normality of distribution with Shapiro–Wilk test and eventual remotion of outliers, a series of parametric correlations were carried out to test: 1) the associations between MUs variables and VO_2_ in any experimental condition, thus resulting in heatmaps, and 2) the hypoxic MUs response (ΔCV and ΔIDR) with hypoxic cardiorespiratory responses (ΔVO_2_, HVR and HCR). Pearson's r coefficients and p values were computed. These analyses were carried out with Prism Version 10 (GraphPad Software, San Diego, USA).

## Results

### Symptoms

Participants reported more symptoms of any nature in mild hypoxia (*n* = 11 out of 18) than in light hypoxia (*n* = 9) and normoxia (*n* = 8), as shown in Table 2.

**Table 2:**
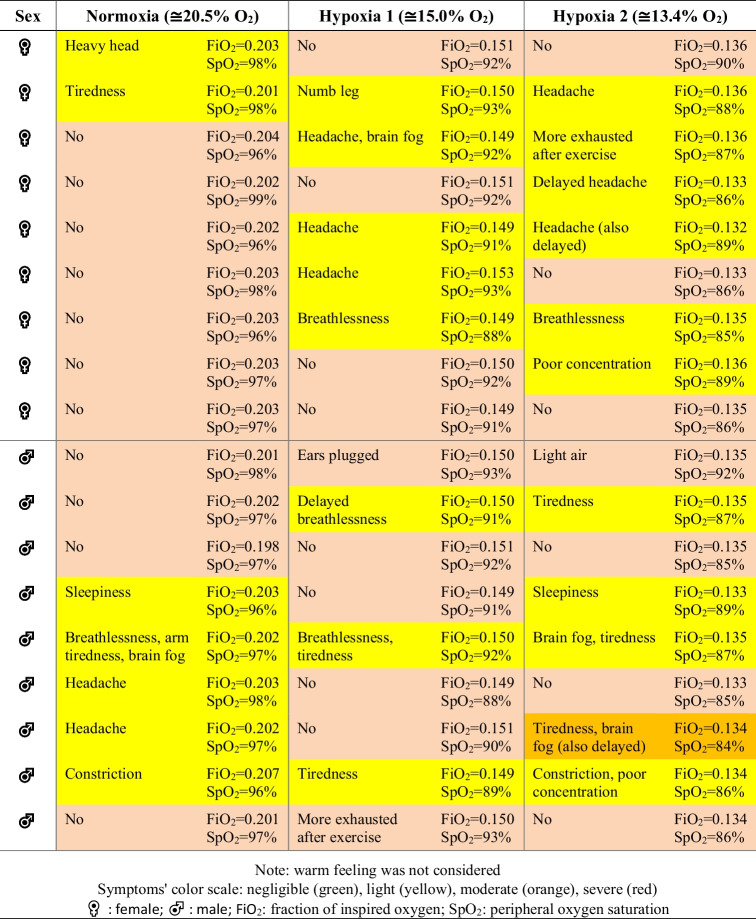
Report of individual symptoms in all experimental conditions

Warm feeling was not considered

Symptoms' color scale: negligible (green), light (yellow), moderate (orange), severe (red)

♀: female; ♂: male; FiO_2_: fraction of inspired oxygen; SpO_2_: peripheral oxygen saturation

### Peripheral saturation

Data obtained from finger pulse oxymeters are shown in Table 3. As expected, SpO_2_ dropped from normoxia to light hypoxia and mild hypoxia (on average, 97.1 ± 0.90%, 91.3 ± 1.60%, and 86.9 ± 1.83%, respectively; RM-ANOVA test resulted in *p* < 0.001 and η^2^_p_ = 0.946).

**Table 3:**
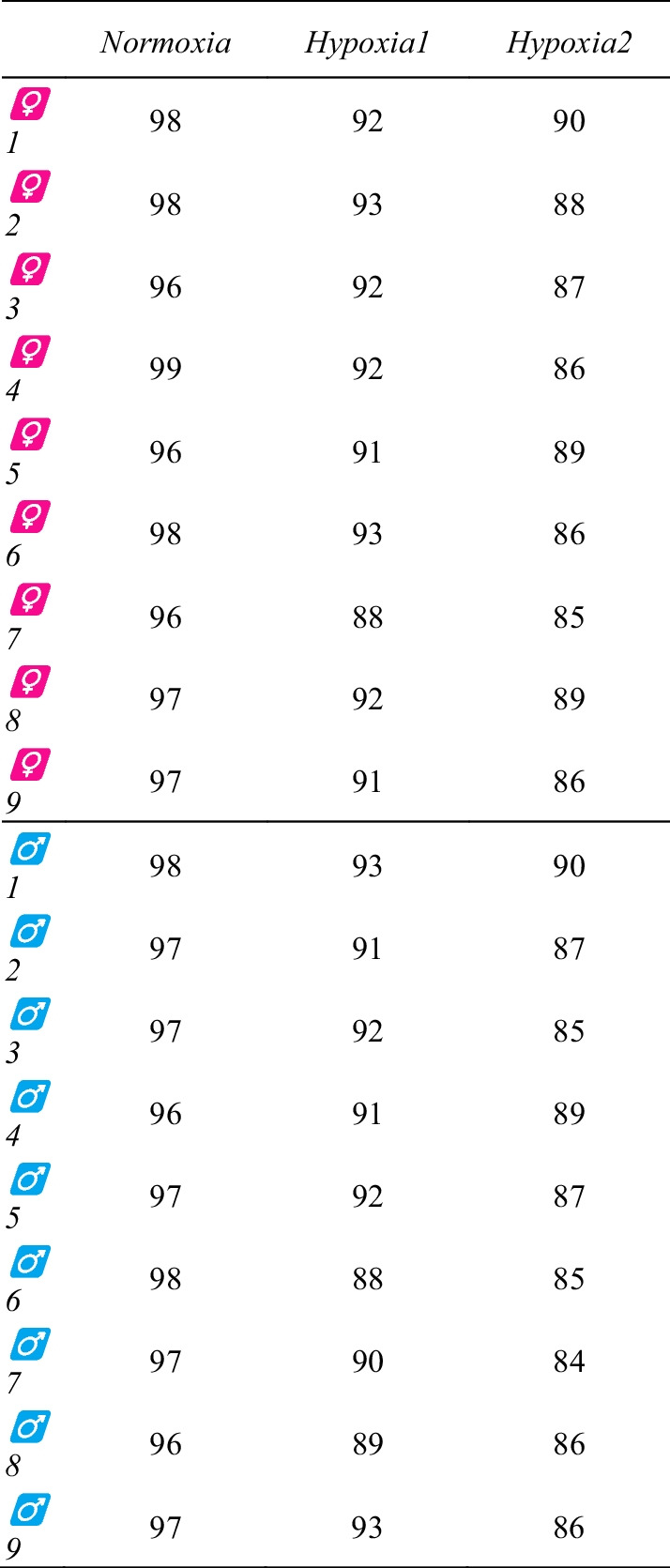
SpO_2_ (%) as registered at the end of each experimental condition

### MUs properties

**Fig. 2:**
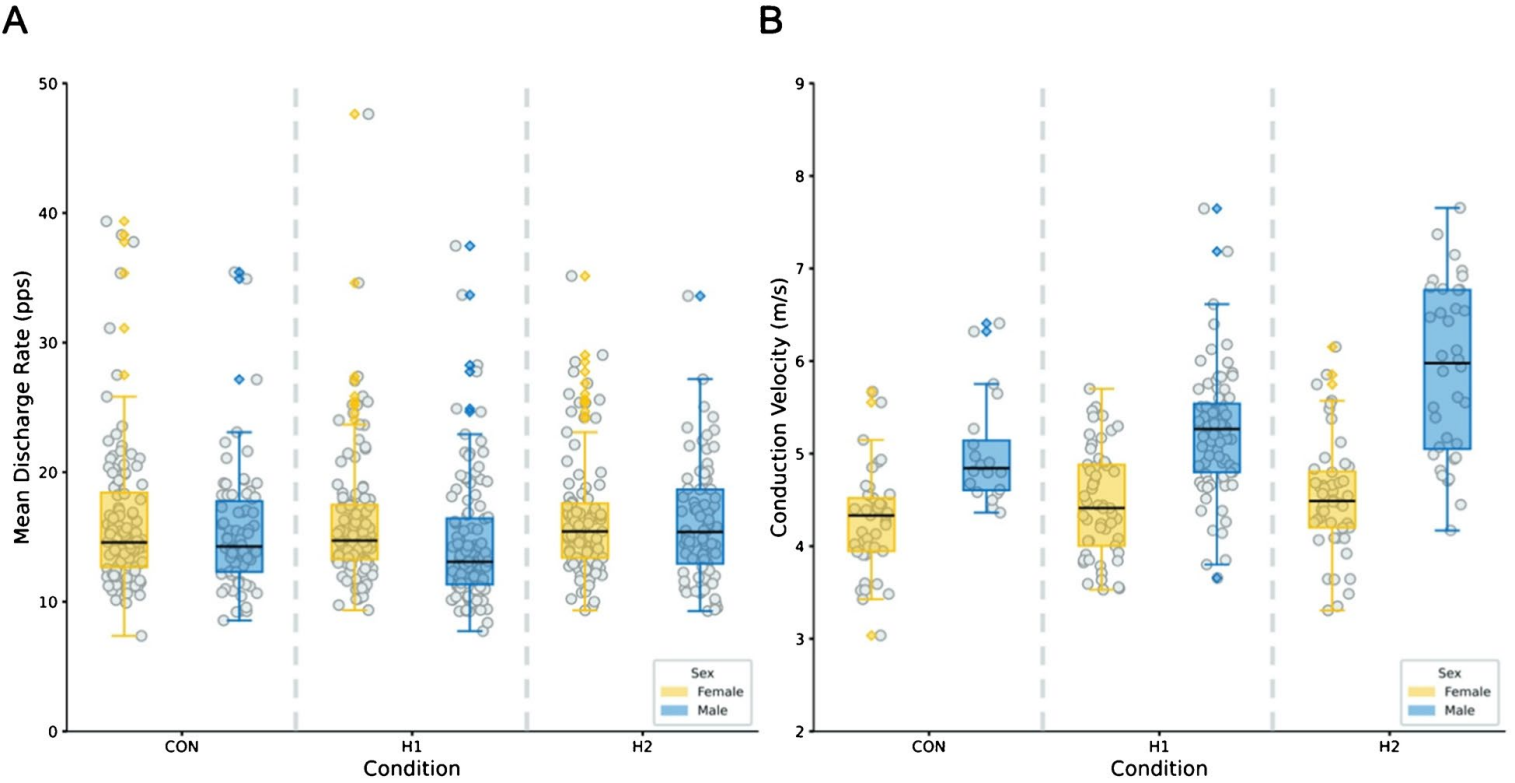
Results of the motor unit (MU) properties analysis. **A** Swarm plot displaying the mean smoothed single MU discharge rate (expressed in pulses per second, pps), accompanied by a box plot illustrating the median values and interquartile ranges across conditions, separately for male and female participants. **B** Swarm plot displaying the single MU conduction velocity (expressed in meters per second), accompanied by a box plot illustrating the median values and interquartile ranges across conditions, separately for male and female participants. CON, control, Normoxia (≅ 20.3% O2); H1, Hypoxia 1 (≅ 15.0% O2); H2, Hypoxia 2 (≅ 13.4% O2)

Interval discharge rate differed across conditions (*p* = 0.010, η^2^_p_ = 0.016, Cohen's *f* = 0.114); it also varied differently across conditions between females and males, although to a lower extent (*p* = 0.042, η^2^_p_ = 0.011, Cohen's *f* = 0.088); specifically, while females experienced an almost linear trend towards greater IDR from control to Hypoxia 2, males had on average minimal values at Hypoxia 1 and maximal at Hypoxia 2, as shown in Fig. 2; raw values of IDR seem lower in males, although the comparison by sex was not statistically significant (*p* = 0.209, η^2^_p_ = 0.097, Cohen's *f* = 0.197). Conduction velocity differed across conditions (*p* < 0.001, η^2^_p_ = 0.070, Cohen's *f* = 0.259) and by sex, with much greater values in males (*p* = 0.001, η^2^_p_ = 0.468, Cohen's *f* = 0.858); both sexes experienced a similar increase from Control to Hypoxia 1 and Hypoxia 2, although non statistically significant (*p* = 0.084, η^2^_p_ = 0.018, Cohen's *f* = 0.106); in particular, the greatest increase was found in males from Hypoxia 1 to Hypoxia 2 (p_Holm_ < 0.001), as shown in Fig. 2. Overall statistical results of linear mixed models are shown in Table 4.

**Table 4:** Statistical results for interval discharge rate (IDR) and conduction velocity (CV) as resulted from linear mixed models fit by restricted maximum likelihood. The scaling used, necessary to show all the data, makes the overall trend in the box lines less visible

	AIC	BIC	Marginal R^2^	Conditional R^2^	Random effect
**IDR**	–1044	–969	0.049	0.314	*p* < 0.001
**CV**	397	426	0.348	0.729	*p* < 0.001

*AIC* Akaike information criterion; *BIC* Bayesian information criterion

### Associations of MUs variables with cardiorespiratory responses

**Fig. 3:**
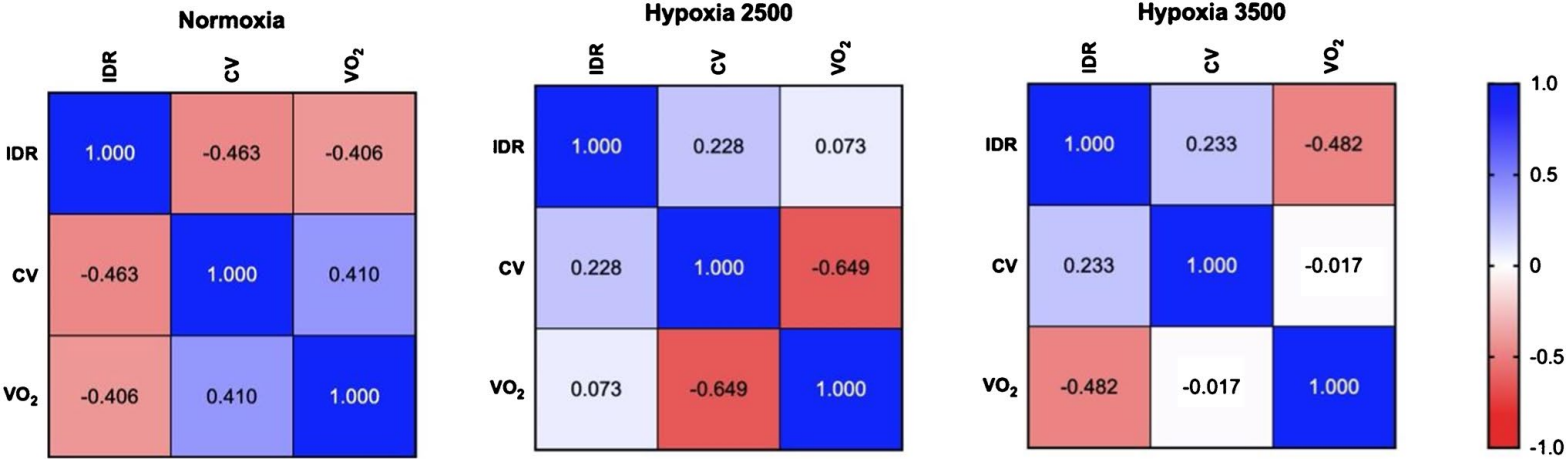
Heatmaps of correlations between MUs variables and oxygen consumption, computed for each of the three experimental conditions; values represent Pearson's coefficients

As shown in Fig. 3, IDR and CV were negatively correlated in normoxic condition (Pearson's r = –0.463), although far from statistical significance (*p* = 0.111), while negligible associations were found during the two hypoxic conditions. During light hypoxia, the higher the VO_2_, the lower the MUs' CV (r = –0.649, *p* = 0.042), while this negative correlation disappeared during mild hypoxia. During normoxia and mild hypoxia, the higher the VO_2_, the lower the MUs' IDR (r = –0.406 and r = –0.482, respectively), although far from statistical significance (*p* = 0.190 and *p* = 0.113, respectively).

The Control – Hypoxia2500 difference of IDR and CV were not correlated (r = –0.175, *p* = 0.587), nor were the Control – Hypoxia3500 difference of these MUs variables (r = 0.196, *p* = 0.563). As shown in Table 5, the difference between normoxia and Hypoxia2500 of both IDR and CV were not associated with VO_2_, nor did those between normoxia and Hypoxia3500. Only slight correlations were found when associating the response of MUs variables and the other cardiorespiratory variables from normoxia to Hypoxia2500. Instead, slight positive correlations, although not statistically significant, were found in the response from normoxia to Hypoxia3500, i.e. CV increment is more linked with HCR than HVR, while IDR increment was linked with both HCR and HVR.

**Table 5:** Correlations between MUs variables (Interval Discharge Rate: IDR, Conduction Velocity: CV) and cardiorespiratory variables (hypoxic ventilatory response: HVR, hypoxic cardiac response: HCR, oxygen consumption: VO_2_) in the response between control condition and the two hypoxic protocols (respectively, Ctrl-Hyp1 and Ctrl-Hyp2)

***Ctrl–Hyp1***		HVR	HCR	ΔVO_2_
ΔIDR	r	0.063	0.082	–0.027
	*p*	*0.845*	*0.801*	*0.933*
ΔCV	r	0.518	–0.043	–0.092
	*p*	*0.234*	*0.927*	*0.845*
***Ctrl–Hyp2***				
ΔIDR	r	0.470	0.521	0.124
	*p*	*0.145*	*0.101*	*0.716*
ΔCV	r	0.608	–0.379	0.223
	*p*	*0.148*	*0.401*	*0.630*

## Discussion

In last decades, the developments of algorithms to decompose surface EMG signals has allowed to obtain information onto constituent trains of MUAP with noninvasive approaches [46]. The advancements in technological devices and signal processing have pushed the development and spread of decomposition algorithms to obtain constituent trains of MUAP from EMG signals, thereby opening new avenues for research into neuromuscular responses to environmental stressors. This field of enquiry is still in its infancy, with a small number of results already published.

Our results showed that both central and peripheral properties of neuromuscular activation are affected by normobaric hypoxia during a series of maximal isometric contraction of *quadriceps*. By using a needle electrode to record intramuscular EMG during a 10% of maximal voluntary contraction (MVC) task, Ruggiero and McNeil [47] reported that lowlanders manifested slightly higher MU discharge rate after being acclimatized to high altitude (≈5,000 m a.s.l.) compared to low altitude (15.2 ± 1.9 vs 13.8 ± 1.5 Hz), and longer MU potential duration. They also reported that, despite a similar whole neuromuscular propagation, the first phase of maximal compound muscle action potential was featured by a lower amplitude and longer duration at high altitude. They interpreted such results as due to putative impairment of sarcolemma excitability and sympathetic-mediated increase of corticospinal excitability at high altitude. Very recently, Pearcey and colleagues [48] reported that acute intermittent hypoxia resulted in greater MU discharge rates in patients with chronic incomplete spinal cord injury, likely via improved motoneuronal excitability and/or synaptic efficacy of excitatory commands.

Our results confirm the putative increase in MU discharge rate as a response to acute normobaric hypoxia during maximal isometric contraction [48], although to a little extent and differently across sexes. This can be due to a plateau in the maximal IDR achievable during maximal contraction in stressful conditions, that limited the possible greater increment due to hypoxia during submaximal contraction.

The increase in MU conduction velocity integrates the current insights by providing evidence for a peripheral enhancement of muscle fiber excitability, thus complementing the presynaptic over-responses triggered by hypoxia. Moreover, very recently Simpson and colleagues reported a normobaric hypoxia-induced increase in type II muscle fibers activation, as estimated by relatively greater spectrum area of high (75–100 Hz, putatively type II fibers) vs low (1–29 Hz) frequency bands [49]. Central motor drive may become relatively more affected at higher degrees of hypoxia, possibly associated with increased muscle fatigue at low frequencies [18]. Males showed greater values of CV than females and experienced a slightly more prominent increase due to hypoxia; such increase was particularly evident in males from Hypoxia 1 to Hypoxia 2. Taking into account the heterogeneity of responses across individuals, we speculate that the hypoxic stress affected differently the neuromuscular system between sex: males slightly reduced IDR and increased CV during light hypoxia, and increased both in mild hypoxia, with a massive increase in CV; collectively, females showed lower responsiveness in MUs properties to hypoxic stress. Recently, similar motor unit activation–deactivation strategies have been reported by comparing males and females during submaximal contraction of *biceps brachii;* in the same study, it was highlighted the higher rate coding in lower‐threshold motor units of females, whilst a greater discharge rate was found in males' higher‐threshold motor units [50]. Hormonal fluctuations, fiber types, ion channels functioning and autonomic nervous system dynamics may be addressed in future studies to depict the sex differences in neuromuscular behaviour while exposed to different stressors.

The central and peripheral features of MUs in normobaric hypoxia during a series of maximal isometric contractions were non-homogeneously linked with oxygen consumption, suggesting that neuromuscular and bioenergetic systems were not entangled during acute normobaric hypoxia of slight to mild extent. It is not excluded that an entangled response with a delay of a system over the other may appear with longer duration of protocols. Indeed, longer protocols or the use of lower FiO_2_ (i.e., more severe normobaric hypoxia) may trigger different integrated responses across physiological systems. During maximal isometric exercise, ventilatory and cardiac hypoxic responses were only slightly associated with hypoxia-induced changes in MUs behaviour. Exercise pressor reflex is due to the mechanoreflex and metaboreflex, and evidence exist on the synergistic or hyperadditive interaction between the carotid chemoreflex and both the exercised muscle reflexes [51]. Here the putative exercise-induced potentiation on HCR and HVR emerged slightly from normoxia to normobaric mild hypoxia, suggesting that the hypoxic dose can trigger over a certain threshold this cardioventilatory-muscle synergistic response.

### Limitations

Inter-individual differences in the dependent variables may be due to the morpho-functional features of muscle systems across participants, as well as to their physical activity level. Indeed, the sports and training history of individuals has been shown to affect differentially the features of high vs low-threshold MUs [52]. Despite the number of participants respected the calculation for the minimum sample size as performed before the study, it is yet not sufficient for dissecting the relative contribution of the mentioned factors on the observed responses. Further studies designed for dissecting the effect of muscle mass, sports disciplines, fitness status on the hypoxic-induced EMG responses are needed. On the same vein, the diversity of symptoms did not allow to evaluate if specific hypoxic-induced symptoms affected MUs properties; however, despite the occurrence of symptoms, muscle strength was shown to be unimpaired during acute hypoxic exposure [53].

In this study, MU properties have been analyzed only during maximal contractions; this was due to the willingness of a pragmatical reproduction of real-life contraction coupled with the absence of an adequate set-up of a lower limb dynamometer into the hypoxic tent, while considering the necessity of standardizing contraction intensity. We cannot exclude that the different training background of participants may have led to potentially greater familiarity with maximal isometric exercise. Moreover, maximal strength may have changed during the series of isometric contraction, due to a plausible fatigability. Although assessing MU properties during maximal muscle force production is the most direct way to assess the influence of hypoxia on the neuromuscular function, MU detection in this condition faces significant challenges [54]. The primary limitation is the increased signal complexity at higher contraction intensities. The recruitment of a larger number of MUs and higher DR result in extensive overlap of MU action potentials, increasing the risk of amplitude cancellation and complicating the accurate separation and detection of individual MUs. Another limitation relies on the absence of ultrasound guidance for determining orientation of the electrode matrix, a common practice for inter-session motor unit tracking. However, we carefully standardized the positioning of the matrix by using a pen-electrode and identifying the muscle belly.

We cannot exclude that limitations of decomposition forced by the experiment design and data collection affected the results. However, due to these limitations, we restricted our analyses to variables less affected by potential errors in MU firing detection (i.e., a smoothed version of the IDR and MU CV). Future studies should focus on assessing MU properties during controlled submaximal contractions, as these may reduce variability, improve detection accuracy, and facilitate the identification of specific responses.

### Perspectives and conclusions

Scholars may leverage our findings to enhance the use of advanced EMG techniques to elucidating the neuromuscular repercussions of hypoxia during exertions that surpass the maximal voluntary contraction typically established in analogous investigations; practitioners could leverage our findings to design hypoxic training regimens, cognizant that the firing rate remains largely unaltered, whereas conduction velocity may exhibit an increase under hypoxic conditions, especially among males.

Answering the question “Can non-invasive motor unit analysis reveal a specific hypoxic-induced response during maximal muscle contraction?”, we can conclude yes, normobaric hypoxia stimulates the neuromuscular system, and the effect reaches a higher extent while observing peripheral rather than central features. However, the slight overstimulation and the great interindividual heterogeneity in response does not allow to affirm sharply that this dose of normobaric hypoxia impairs maximal force production by disturbing MUs function.

## Supplementary Information

Below is the link to the electronic supplementary material.

Supplementary file1 (DOCX 32 KB)

## Data Availability

No datasets were generated or analysed during the current study.

## Declarations

### Generative artificial intelligence (GAI)

No GAI tool was used in the preparation of this manuscript.
