# Supplementary material for: Non-invasive motor unit analysis reveals specific responses during maximal muscle contraction under normobaric hypoxia
Source: Pflugers Arch. 2025 Sep 10;477(10):1295–308. doi: 10.1007/s00424-025-03119-y (PMC12488839; doi:10.1007/s00424-025-03119-y)
Supplement: Supplementary file 1 — Supplementary file1 (DOCX 32 KB) [file 424_2025_3119_MOESM1_ESM.docx]

**Supplementary materials - Bioimpendance anlaysis**

*Bondi, Valli, Santangelo, Annarumma, Pietrangelo, Fulle, Verratti*

Anthropometry was conducted by measuring the weight and height to the nearest 0.1 kg and cm accuracy, using the Seca 707 digital balance, properly calibrated, and the Seca 213 stadiometer (Seca GmBH & Co Kg, Hamburg, Germany). Anthropometrics data were used to assess the body mass index (BMI, as m/h^2^). Body composition measurements were carried out by a specialist using the HUMAN IM TOUCH multi-frequency analyzer (DS MEDICA, Milan, Italy). The Body Impedance Analysis (BIA) is based on electrical properties that in biological system intra and extra-cellular electrolytic solutions contribute to the resistance (R), and the integrity of the cell membranes acting as a capacitor contributes to the capacitive reactance (Xc) (Khalil *et al.*, 2014). The BIA device measures the resistance (Impedance- Z) of all tissues to an injected high-frequency (from 5 kHz to 250 kHz), low-amplitude alternating electric current (500 to 800 mA). The delay in the current flow and the lag caused by the storage of electrical energy on healthy cell membranes creates a phase difference between the current and voltage, which is expressed as the phase angle (PhA), specifically the arc-tangent value of the ratio of Xc to R. This factor provides indication for cellular health in terms of electrical integrity of vital cell membrane and cell function (Sardinha, 2018). Higher values correspond to better cellular integrity and muscle quality; useful PhA cut-off values for young males and females subjects are respectively 5.95° and 5.02°, up to 9° in ideal physical conditions (Raschka *et al.*, 2020; Akamatsu *et al.*, 2022). The BIA software provides predictive equations which includes all these raw parameters, along with variables such as age, stature, and weight, allowing to estimate: total body water (TBW), intra and extracellular water (ICW and ECW), fat mass (FM), fat-free mass (FFM), fat mass index (FMI), free fat mass index (FFMI), body cellular mass (BCM), skeletal muscle mass (SM), and skeletal muscle mass index (SMI). In this study, the predictive equation was chosen among those included in the software for each subject based on the bioelectrical values and on the software indication. Specifically, in the current study, BIA parameters were employed to assess and profile the physical characteristics of the participants considering PhA (50 kHz), TBW %, FM%, SMI; and SMIleg%, SM and SMleg. During BIA measurements, current was injected through electrodes—patch placed on the anterior surface of the skin; the two injector electrodes were located in the metacarpophalangeal and metatarsophalangeal joints; the sensing electrodes were placed at the wrist and ankle. In addition to whole body measures, we implemented regional BIA measurement through the use of two electrodes on contralateral wrist and ankle. The acquisition was conducted in a supine position maintained for 5 min before the test, with legs and arms slightly abducted (respectively ∼45◦ and 30◦) to avoid contact between body segments. Any of the standard conditions for the measurements were met (empty bladder, cleaned skin, last beverages consumption, exercise at least 12 h before the measurement, and fasting state >3 h (Walter-Kroker *et al.*, 2011) .

Data obtained from BIA are shown in **Table 1** (main text). Anthropometrics were used to assess the Body Mass Index, revealing that the participants were within the reference range for normal weight (BMI 18.5-24.9 kg/m^2^), except for three individuals who were slightly overweight (up to 29.9 kg/m^2^). One male participant showed particularly high BMI (35.1 kg/m^2^), usually class II obesity, but attributed to high muscularity due to his advanced level of sport and fitness, and therefore he has been excluded from the bioimpedance analysis. PhA was found in line with the reference cut-off for both sex, suggesting optimal musculoskeletal and fitness status of our participants, also considering the association of PhA with handgrip strength and explosive strength of the lower limbs (Ballarin *et al.*, 2024). Skeletal muscle mass was evaluated considering its normalization by height as SM index (SMI = SM/h^2^). SMI was also showed as positive predictor for lifted maximum load at one repetition during resistance training (Sue *et al.*, 2022). The cut-off points useful for defining the absence of muscle quality and quantity impairments are SMI ≥ 10.76 kg/m^2^ and ≥ 6.76 kg/m^2^ respectively in men and women (Gonzalez & Heymsfield, 2017). SM was also registered for lower limbs (SMleg), then computing SMI for lower limb (SMIleg) and reporting SMleg as percent values of whole SM.

**References (all of which are included in the main text as well)**

Akamatsu Y, Kusakabe T, Arai H, Yamamoto Y, Nakao K, Ikeue K, Ishihara Y, Tagami T, Yasoda A, Ishii K & Satoh-Asahara N (2022). Phase angle from bioelectrical impedance analysis is a useful indicator of muscle quality. *Journal of Cachexia, Sarcopenia and Muscle* **13,** 180–189.

Ballarin G, Valerio G, Alicante P, Di Vincenzo O, Monfrecola F & Scalfi L (2024). Could BIA-derived phase angle predict health-related musculoskeletal fitness? A cross-sectional study in young adults. *Nutrition* **122,** 112388.

Gonzalez MC & Heymsfield SB (2017). Bioelectrical impedance analysis for diagnosing sarcopenia and cachexia: what are we really estimating? *J Cachexia Sarcopenia Muscle* **8,** 187–189.

Khalil SF, Mohktar MS & Ibrahim F (2014). The theory and fundamentals of bioimpedance analysis in clinical status monitoring and diagnosis of diseases. *Sensors (Basel)* **14,** 10895–10928.

Raschka C, Koch HJ & Käsebieter J (2020). Change of hydration parameters in moderate altitude: measurements in healthy volunteers by means of bioelectric impedance analysis. *Papers on Anthropology* **29,** 40–49.

Sardinha LB (2018). Physiology of exercise and phase angle: another look at BIA. *Eur J Clin Nutr* **72,** 1323–1327.

Sue K, Kobayashi Y, Ito M, Midorikawa-Kijima M, Karasawa S, Katai S & Momose K (2022). Bioelectrical impedance analysis to estimate one-repetition maximum measurement of muscle strength for leg press in healthy young adults. *Sci Rep* **12,** 17142.

Walter-Kroker A, Kroker A, Mattiucci-Guehlke M & Glaab T (2011). A practical guide to bioelectrical impedance analysis using the example of chronic obstructive pulmonary disease. *Nutr J* **10,** 35.
